# Cancer Incidence Characteristic Evolution Based on the National Cancer Registry in Taiwan

**DOI:** 10.1155/2020/1408793

**Published:** 2020-07-22

**Authors:** Yu-Ching Huang, Yu-Hung Chen

**Affiliations:** School of Medicine, College of Medicine, Fu Jen Catholic University, New Taipei, Taiwan

## Abstract

**Introduction:**

Taiwan has committed itself to cancer prevention. This study investigates the impact of cancer prevention on cancer incidence in Taiwan.

**Objective:**

This study describes the secular trends and present status of cancer incidence in Taiwan during the years of 1988 to 2016.

**Methods:**

Age-standardized incidence rates (ASRs), age-specific incidence, and sex ratios for all cancers were calculated using data from the Taiwan Cancer Registry System for the years 1988 to 2016. *Results and Conclusions*. ASRs of cancer for males increased from 150.93 per 10^5^ individuals in 1988 to 330.03 per 10^5^ individuals in 2016, and, for females, they increased from 124.18 per 10^5^ individuals in 1988 to 269.5 per 10^5^ individuals in 2016. We found that cancer incidence has begun at younger ages and that the rates of cancer incidence are increasing faster. This study shows that the incidence of cancer in males has decreased slightly in recent years, while the incidence of cancer in females has continued to increase. The continuous promotion of health literacy, lifestyle modification, HBV and HPV vaccination, and cancer early screening can improve the effectiveness of cancer prevention.

## 1. Introduction

Cancer is becoming an increasingly important public health problem in Taiwan due to an aging population, as well as environmental and lifestyle changes. Not only does cancer cause harm to the patient, but also treatment costs and long-term treatment courses also place significant burden on families and caregivers. In 1984, the Taiwanese government launched a nationwide HBV vaccination program for all newborn infants [1]. After the launch of the National Health Insurance (NHI) in 1995, a subsequent national screening program for breast cancer, cervical cancer, colorectal cancer, and oral cancer was launched in 2004 [2]. In Taiwan, most cancer treatments are reimbursed by the NHI's Catastrophic Illness program [3]. If a patient has a Catastrophic Illness Card, he or she will be exempt from copayments for outpatient visits and hospitalizations [4]. However, these medical expenses also impose a large burden on the society [2], with the proportion of cancer treatment expenses within NHI expenditures rising from 5.57% in 1999 to 10.96% in 2017.

Multiple concerted efforts in cancer prevention, screening, treatment, and palliative care have been enacted in the last few decades [5]. Taiwan's Cancer Control Act was enacted in 2003 and last amended in 2018; the Hospice and Palliative Care Act was enacted in 2000 and last amended in 2013; the Hospice Palliative Care Act was enacted in 2013; and the Tobacco Hazards Prevention Act was enacted in 2009 [6]. Moreover, three phases of the National Cancer Control program have been implemented since 2005, with the 4^th^ phase starting in 2019. In addition, the policies stipulate that in order to build a database related to cancer control, medical institutions involved in cancer control should submit diagnostic information from cancer patients to academic research institutions commissioned by the governing authorities [7].

It is estimated that by 2018, Taiwan will have crossed the threshold into an aged society (with an elderly population growing to 14%) [8]. Aging is, by far, the most important risk factor for cancer [9]. Therefore, we aimed to examine the long-term trends in cancer incidence in Taiwan, and how the combination of carcinogenic factors and an aging society impacts cancer incidence among different cohorts. As the NHI covers 99% of the Taiwanese population, examining all cancer sites allows us to comprehensively understand changes in the overall medical environment [10]. Therefore, this study describes the secular trends and present status of cancer incidence in Taiwan during the years of 1988 to 2016.

## 2. Materials and Methods

### 2.1. Sources and Data Quality

Data on age-specific cancer incidence was abstracted from the Taiwan Health Promotion Administration's (Ministry of Health and Welfare, MOHW) Interactive Online Cancer Registry Inquiry System [11]. Classification of cancer sites was according to the International Classification of Diseases for Oncology (ICD-O). The Taiwan Cancer Registry (TCR) is a population-based national cancer registration system that was established by the Ministry of Health and Welfare in 1979. The TCR has a coverage rate of 98.4% and accounts for 0.9% of death certiﬁcate only (DCO). The percentages of histological and morphological veriﬁcation (MV) among all cancer sites were 93.0% and 97.6% after excluding the liver [12].

### 2.2. Statistical Analysis

We used the 2000 world standard population [13] to calculate age-standardized incidence. The average annual percent change (AAPC) for the last 30 years was assessed according to the model proposed by Clegg et al. [14]. We used age groups ranging from 0–4 to 85+ years old, a total of 18 groups. The years ranged from 1988 to 2016, split into five-year groups, and included 1988–1992, 1993–1997, 1998–2002, 2003–2007, 2008–2012, and 2013–2016, for a total of six groups, with the last group only containing four years. Birth cohorts were divided into a total of eight groups.

## 3. Results

**Figure 1:**
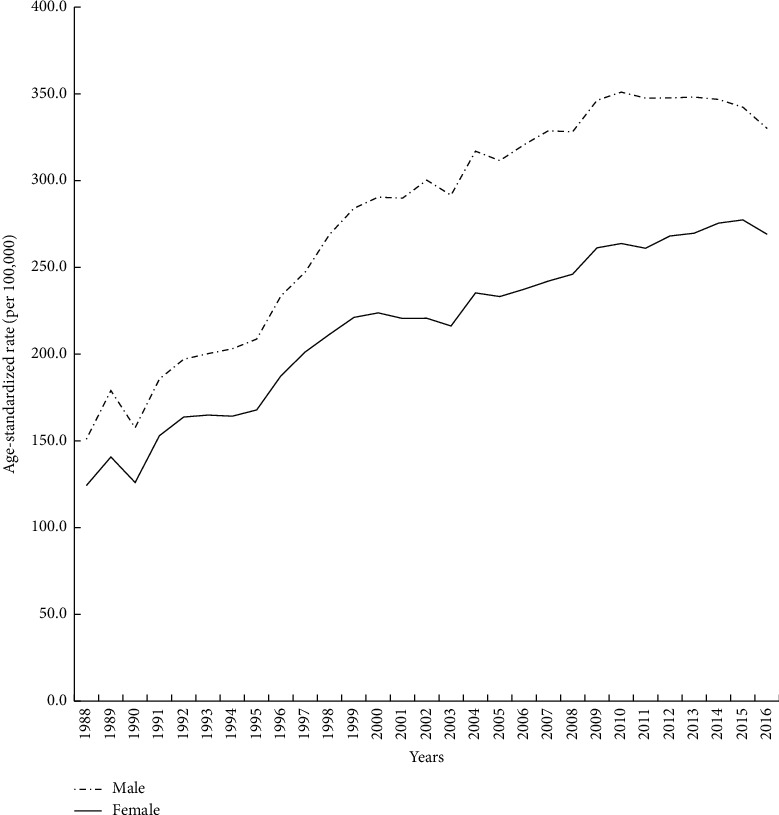
Secular trend in age-standardized incidence rates of all cancers in Taiwan, 1988–2016.

Figure 1 shows the secular trends in age-standardized rates of all cancers in men and women in Taiwan, from 1988 to 2016. For males, the age-standardized incidence per 100,000 individuals increased from 150.93 per 10^5^ individuals in 1988 to 330.03 per 10^5^ individuals in 2016 for an AAPC of 3.01%. This indicates that the age-standardized incidence increased by 7.38 per 10^5^ individuals every year. For females, the age-standardized incidence increased from 124.18 per 10^5^ individuals in 1988 to 269.05 per 10^5^ individuals in 2016, for an AAPC of 2.96%, meaning that the age-standardized incidence increased by 5.40 per 10^5^ individuals every year.

**Figure 2:**
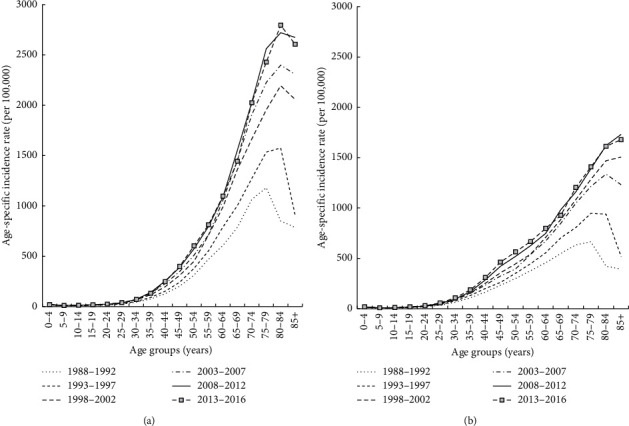
Age-specific incidence rates of all cancers by calendar year: (a) males and (b) females.

Figure 2(a) shows that the age-specific incidence in men between 1988 and 2016 increased steadily starting from 0–4 years of age, peaking after 75 years of age. Among the same age group, more recent time periods had higher cancer incidence than older time periods. Moreover, for each time period, the age-specific incidence increased at a higher rate in males than in females. As can be seen from Figure 2(b), the age-specific incidence in women was generally lower than in men, and the age at which the highest cancer incidence occurs increased with more recent time periods.

**Figure 3:**
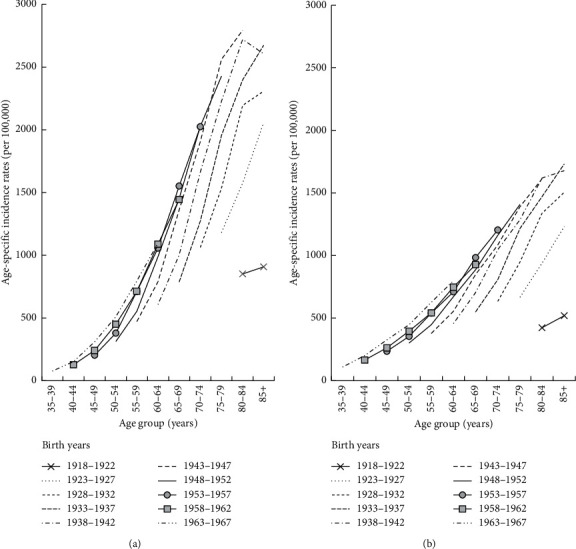
Age-specific incidence rates of all cancers by birth year and sex: (a) males and (b) females.

Figure 3 shows age-specific incidence rates in males and females according to birth cohort. For both males and females, younger cohorts had higher rates of cancer incidence than previous older birth cohorts. For example, at 65–69 years of age, men born in 1953–1957 had incidence rates that were 97.30% higher than those born in 1933–1937, while the difference in women for these same two birth cohorts was 79.32%. Within the same birth cohort, the cancer incidence in males was higher and increased faster than in females.

Table 1 shows that the sex ratio for age-standardized incidence rates of all cancers across all age groups was between 1.23 and 1.35. We found that the sex ratios for ages 0–19 years and 50–85 years were greater than 1, and that the sex ratios for ages 20–49 years were less than 1. Sex ratios declined with age between 0 and 29 years and then gradually increased with age, beginning to decrease again after ages 75–79.

Table 2 shows the changes in incidence and rank of major cancers affecting the Taiwanese population. Among males, liver cancers accounted for 10.11% of all cancers during 1988–1992, increased to 18.98% in 1998–2002, and then decreased to 14.01% in 2013–2016. The mean age of liver cancer cases increased from 56.83 years in 1988–1992 to 63.46 years in 2013–2016 in males and from 59.61 years to 69.77 years in females. In addition, the proportion accounted for by lung cancer increased from 8.55% in 1988–1992 to 14.68% in 1993–1997 and decreased to 13.33% in 2013–2016 in males. On the other hand, the proportion increased from 3.37% in 1988–1992 to 11.18% in 2013–2016 in females. In 1988–1992 and 2008–2012, the mean age of lung cancer cases increased from 64.51 years to 69.28 years in men and increased from 62.14 years to 66.22 years in women. However, the mean age decreased to 68.34 years in males and 65.86 years in females in 2013–2016.

The percentage of men with colorectal cancer increased from 6.44% in 1988–1992 to 15.71% in 2013–2016, and, in women, it increased from 4.81% in 1988–1992 to 14.40% in 2008–2012 and then decreased to 13.98% by 2013–2016. Moreover, the mean age in men and women in 1988–1992 was 61.35 years and 60.20 years, respectively, and in, 2013–2016, it was 65.92 years and 66.12 years, respectively.

From 1988–1992 to 1998-2002, gastric cancer, accounting for 6.22% of all cancers, increased to 6.91% and decreased to 4.17% by 2013–2016 in males. Among females, the proportion increased from 2.71% in 1988–1992 to 5.51% in 1993–1997 and decreased to 2.99% by 2013–2016, and the mean age increased from 59.22 years in 1988–1992 to 67.78 years in 2013–2016. In addition, among male-specific cancers, the proportion of prostate cancer increased from 1.85% to 8.96%, and its ranking increased from eighth to fifth. Among women, cervical cancer was ranked first in 1998–1992, accounting for 7.95% of all cancers, but fell to eighth place in 2013–2016, accounting for 3.12%. In addition, breast cancer was ranked second in 1988–1992 and ranked first in 1993–1997 to 2013–2016. However, while it only accounted for 6.54% in the first time period, its proportion increased significantly to 25.26% by 2013–2016. The proportion for female thyroid cancer increased from 1.50% in 1988–1992 to 5.49% in 2013–2016. The mean age of thyroid cancer cases was 42.08 years and 48.97 years in 1988–1992 and 2013–2016, respectively.

## 4. Discussion

The results from this study showed that the incidence of the most important cancers in Taiwan is increasing, including rates of lung, thyroid, breast, and prostate cancer. The factors contributing to these increases in incidence are the following: first, the coverage rate of the NHI being increasing [10]; second, the launch of national oral cancer, colorectal cancer, breast cancer, and cervical cancer screening programs; third, increased exposure to environmental pollution [15]; fourth, changes in lifestyle habits; and, the last, reduction in deaths from competitive risks. However, due to concerted public health efforts in cancer prevention and control, there has been a decline in the incidence of some cancers. Factors contributing to the decline in these cancers include the following: first, long-term screening programs, such as cervical cancer; second, long-term availability of vaccination or therapy programs for certain cancers, such as liver cancer; and, third, changes in lifestyle and nutritional habits, which affect cancers such as stomach cancer and male squamous cell lung cancer.

Looking at the secular trends of cancer incidence in Taiwan from 1988 to 2016, age-standardized rates in males and females increased faster than in other countries. In particular, the AAPC in Taiwanese males during 2003 to 2012 was 1.53%, from 291.48 per 10^5^ individuals to 347.58 per 10^5^ individuals, and, in Taiwanese females, it was 2.01%, from 216.18 per 10^5^ individuals to 267.99 per 10^5^ individuals. In contrast, the AAPC in American males during 2003 to 2012 was -1.4%, and, in American females, it was 0.0%. [16] However, the slope of the secular trend in age-standardized incidence rates of all cancers was decreasing because of the national major cancer screening programs from 2004 [2]; the incidence of young people was stable gradually. We also found that the sex ratios of incidence in those aged 20–49 years were less than 1. Moreover, the increase in cancer incidence among women being born in 1968–1972 was higher than in other age groups in the past 30 years; this may be because cancer screening has significantly impacted female cancers. For example, the incidence of breast, cervical, and thyroid cancer, which are major cancers in women, increased most likely due to the implementation of increased screening and medical surveillance, leading to increased early detection. On the other hand, the cancer incidence in males aged 80–84 years was greater than in women of the same age, and the sex ratio for individuals between 50 and 85 years old was greater than 1. Changes in cancer incidence among males were concentrated in the elderly, as most of the major cancers in men occur in old age, such as prostate, colorectal, and liver cancer.

Looking at birth cohorts, among both sexes, younger generations had a higher incidence of cancer than older generations of the same age group. Factors affecting an increase in cancer incidence may have included the implementation of screening programs for cancers such as oral, colorectal, breast, and cervical cancer. Environmental changes over the years have also increased chances of being exposed to carcinogenic factors. For example, in the 1950s, the Taiwanese government implemented a program to eradicate malaria that included the large-scale use of DDT residual spray. A study by Chang et al. later showed that women who were born between 1951 and 1959 and exposed to DDT during childhood had a subsequent increased incidence of breast cancer [17].

The incidence of female breast cancer increased from 20.95 per 10^5^ individuals in 1988–1992 to 71.91 per 10^5^ individuals in 2013–2016. This increase may be attributable to various factors, including hormonal and reproductive risk factors such as early menarche, late menopause [18], low parity, older age at first live birth [19], low prevalence of breastfeeding [20], high-fat intake, alcohol consumption, and low levels of physical activity. In addition, exposure to excessive environmental hormones also affects the secretion of estrogen [21]. Moreover, Taiwan launched a national breast cancer screening program in 2002, with an ability to detect very early stage cancers; even a low screening acceptance rate of 17% [19] has led to a significant increase in incidence.

Thyroid cancer occurs more often in young women because it is easier to diagnose papillary carcinoma (PTC) in women than in men [22], and PTC accounts for 89% of thyroid cancers. The age-standardized rate in women increased from 4.39 per 10^5^ individuals in 1988–1992 to 17.29 per 10^5^ individuals in 2013–2016, due to the introduction of new diagnostic techniques such as ultrasound, tomography, and nuclear magnetic resonance [23]. In Taiwan, this resulted in the detection of a large number of small thyroid nodules [24], which also contributed to the increasing age-standardized rate. Although thyroid cancer has a high incidence rate, the mortality rate is relatively low, showing almost no increase from 1995 to 2017 at 0.28 per 10^5^ individuals [24]. A similar situation has also been seen in Korea [25].

Smoking is the leading cause of lung cancer [26], and both Eastern and Western countries have been committed to reducing smoking rates. Since the enactment of the Smoking Prevention and Control Act in 1997, the smoking rate in men over 18 in Taiwan dropped from 59.4% in 1990 to 40% in 2005 [27], which led to a reduction in the incidence of squamous cell carcinoma caused by smoking. The annual incidence in males dropped from 10.9 per 10^5^ individuals to 10.5 per 10^5^ individuals, and, in females, it dropped from 2.1 per 10^5^ individuals to 1.6 per 10^5^ individuals [28]. The rate of smoking cessation among Taiwanese men increased with age; therefore, the mean age of carcinoma cases only increased by 3.83 years [29]. However, the disease pattern of lung cancer has changed. An increasing incidence of lung adenocarcinoma has caused an increase in the age-standardized rate of lung cancer. From 1996 to 2008, the rate in males increased from 9.9 per 10^5^ individuals to 18.5 per 10^5^ individuals, and, in women, it increased from 7.6 per 10^5^ individuals to 16.3 per 10^5^ individuals [28]. The percentage of lung adenocarcinoma also increased with age, particularly in females in Taiwan, which increased from 53.9% in 1996–1999 to 70.9% in 2000–2004 [28]. In addition to environmental factors such as long-term exposure to PM2.5 [30] and inhalation of kitchen fumes [31], this increase may also be associated with higher genetic susceptibility to EGFR among nonsmoking women in Asia [32].

The incidence of oral cancer in men increased from 10.86 per 10^5^ individuals in 1988–1992 to 42.72 per 10^5^ individuals in 2013–2016. As many Taiwanese men have the habit of smoking cigarettes, drinking alcohol, and chewing betel nuts, their chances of exposure to carcinogens were greatly increased [33]. In 2004, Taiwan launched a national oral cancer screening program [2]. Later studies found that by using visual inspection plus pathological diagnosis, the sensitivity and specificity for detecting oral cancer were 98.9% and 98.7% [34], respectively. However, overall in 2004–2009, the screening rate was only 55.1% [35]. Therefore, encouragement to quit bad habits such as smoking cigarettes, drinking alcohol, and chewing betel nuts and the promotion of regular screening is expected to reduce the incidence of oral cancer in the future [36].

The incidence rate of prostate cancer has increased sharply in Taiwan. According to Pu's research, it is due to the rapid aging of the Taiwanese population [37]. The life expectancy for males in Taiwan increased from 70.99 years in 1988 to 77.53 years in 2018, and the elderly population ratio reached 14.1% in 2017 [38]. Factors contributing to an increased incidence include the routine provision of prostate-specific antigen (PSA) and ultrasonic clinical screening by the National Health Insurance, as well as an increase in high-fat diets [37].

The incidence of colorectal cancer in both sexes has increased significantly over the study period, and the prevalence rates of colorectal hyperplastic polyps and adenomatous polyps in Taiwan were 11.1% and 16.1%, respectively [39]. As food and lifestyle habits changed, including reduced exercise, increased meat consumption and alcohol consumption [40], and increased life expectancy, the possibility of developing cancer was increased. In 2004, Taiwan launched a national colorectal cancer screening program that provided fecal immunochemistry tests (FIT) [41], and, in 2013, it began subsidizing fecal occult blood tests (FOBT). Biennial regular screenings combined with colonoscopy [42] are expected to reduce the incidence of colorectal cancer in the future [43].

Surprisingly, the age-standardized incidence rate of cancer in males declined in 2011–2016, and this decline was mainly due to liver cancer, stomach cancer, and squamous cell lung cancer [28]. Starting in the 1988–1992 time period, the age-standardized incidence rate of male liver cancer in Taiwan gradually increased from 30.52 per 100,000 and reached its peak in 2007 at 56.99 per 100,000. Rates have declined ever since. Taiwan launched a neonatal hepatitis B immunization program in 1984, reducing the prevalence of chronic HBV infection from 9.7% among college students born before 1974 to less than 1.0% in those born after 1992 [44]. On top of that, the national HBV therapy launched in November 2003 may reduce the risk of infant fulminant hepatitis (IFH), chronic liver disease (CLD), and hepatocellular carcinoma (HCC) [45]. Among 1509 patients, which were 6–26 years old, diagnosed with HCC between 1983 and 2011, the incidence rate of HCC was 0.92 per 10^5^ person-years in the unvaccinated birth cohort and 0.23 per 10^5^ person-years in the vaccinated cohort [46]. Moreover, the use of direct-acting antiviral drugs (DAA) has increased the virus clearance rate from 30% to 70% [47]. In addition, female liver cancer increased from 8.72 per 10^5^ individuals in 1988–1992 to a peak of 22.77 per 10^5^ individuals in 2003–2007, after which rates began to decline. Interestingly, the mean age of women with liver cancer in 2013–2016 was 6.31 years higher than that of men, presumably because hepatitis C progresses more slowly in women [48]. A study by Su et al. stated that as vaccine protected cohorts will reach 50 years of age in 2035, a further significant reduction in adult liver cancer incidence in Taiwan will occur, and, by 2035, incidence rates will have decreased by 37.3% in men and 27% in women from 2004 rates [49]. However, as lifestyle changes continue and incidence of fatty liver, obesity, hyperlipidemia, and diabetes caused by alcoholic liver disease (ALD) or metabolic syndrome (MS) continue to increase [50], this has also led to an increase in risk for nonviral liver cancer [51]. Therefore, maintaining good health behaviors and reducing alcohol consumption may help to prevent nonviral liver cancers [52].

The age-standardized rate of cancer in females is still increasing due to breast cancer and lung cancer; however, the cervical cancer is decreasing. The age-standardized rate of cervical cancer decreased from 26.27 per 10^5^ individuals in 1988–1992 to 8.72 per 10^5^ individuals in 2013–2016. Taiwan launched an annual cervical cancer screening program in 1995 [53]. Annual Pap smear screening rates increased from 9.4% in 1995 to 27.5% in 2007, and the three-year screening rates increased from 33.9% in 1997 to 51.0% in 2007 [54]. From 2003 to 2015, the prevalence of regular screenings every three years was approximately 54% [55]. The reasons for low screening rates include women's fear of discomfort or pain, shyness, lack of medical knowledge, or busyness at work [56]. This has resulted in a large number of women being diagnosed with advanced cervical cancer who had not been screened regularly [57]. Thus, the mean age of invasive cancer cases increased from 53.18 to 57.83 years.

The age-standardized incidence rate of stomach cancers among both males and females decreased, mainly due to changes in lifestyle habits. The growing popularity of refrigerators improved the preservation of food, increased the supply of fresh fruits and vegetables, and reduced dependence on preserved foods [58]. Moreover, the launch of a *Helicobacter pylori* infection therapy program decreased the prevalence of *Helicobacter pylori* infection and increased antibiotic therapy in infected patients [59].

The findings of this study should be interpreted with caution in light of the following limitations. First, the accuracy of cancer incidence numbers may have evolved over time due to advances in diagnostic techniques, potentially rendering some incidence rates incomparable between different time periods. Second, the ICD-9 coding scheme in the Taiwan Cancer Registry was replaced with the ICD-O edition in 2002, which may have influenced the changes in diagnostic coding. Third, we focused on trends of all cancer incidence; the cancer control effects evaluated in this study reflect early stage medical treatment, so we are unable to discuss cancer survival rates and mortality associated with middle and late stage medical treatment. In addition, ethnicity-based cancer registration data may not include all clinical or policy variables that account for the results of this study, so caution should be exercised in interpreting the data. However, the results of this study can be provided to government agencies as a reference for cancer surveillance and future cancer control policies.

According to the long trend of the age-standard rate of major cancers, the incidence of some cancers has been declining, such us nasal pharyngeal cancer in both sexes, stomach cancer in both sexes, and cervical cancer. The incidence of cancers that increased first and then decreased includes lung cancer in male and sexual cancers, which were colorectal cancer, liver cancer, and bladder cancer. However, the incidence of oral cancer, esophageal cancer, and prostate cancer in males is increasing, and, for females, lung cancer, thyroid gland cancer, breast cancer, ovarian cancer, and uterine corpus uterus cancer are increasing. In terms of cancer control, the incidence of some cancers has been declining, while not in others. However, we are optimistic for the future. It is critical that the government continues to promote the importance of regular cancer screening. For example, screening for cervical cancer has already resulted in declining incidence. In addition, the government has also implemented a publicly funded HPV vaccine program for girls aged 9–14, in accordance with the recommendations of the World Health Organization (WHO). Although the incidence of breast cancer has not decreased, continuous screening will reduce the prevalence pool, and we expect that the incidence will decline in the future [60]. The disease type of lung cancer among women is also changing, and a study by Lin et al. showed that a risk-based prediction model based on family history of lung cancer and female sex can potentially improve the efficiency of lung cancer screening programs in Taiwan [61]. The incidence of prostate cancer is also increasing. The government should examine its funding resources and consider whether to include these two cancers in their screening programs. Moreover, clinical guidelines need to be modified to avoid overdiagnosis, such as in thyroid cancer. In addition, the government can promote health awareness through health education, so that the incidence of cancers such as liver cancer, which is increasingly caused by metabolic problems, gastric cancer, which is caused by poor eating habits, and cancers caused by smoking, such as lung, oral, cervical, and liver cancers, will eventually decline. If the government promotes early detection and health literacy, as well as various public health strategies to prevent cancer, such as DNA screening to HPV, low-dose computed tomography (LDCT) scanning of lung cancer, the decline in cancer incidence in the future will be due to decreases in cancer among young people.

## 5. Conclusion

Our study showed decreasing cancer incidence potential in major cancers among the Taiwanese population. Besides, the promotion of health literacy, lifestyle modification, improving HBV and HPV immunization to overcome cancer risk factors, and integration of screening strategies could improve the effectiveness of cancer incidence. This study provided a valuable reference for governmental strategies to cancer prevention.

## Figures and Tables

**Table 1:** Sex ratios of age-standardized cancer incidence rates in Taiwan.

Periods	Age (years)																		
	0–4	5–9	10–14	15–19	20–24	25–29	30–34	35–39	40–44	45–49	50–54	55–59	60–64	65–69	70–74	75–79	80–84	85+	Total
1988–1992	1.26	1.16	1.02	1.04	0.87	0.72	0.68	0.71	0.77	0.87	1.04	1.25	1.33	1.44	1.68	1.77	2.01	2.00	1.23
1993–1997	1.22	1.15	1.25	1.18	0.77	0.66	0.68	0.69	0.76	0.93	1.08	1.24	1.42	1.43	1.58	1.62	1.68	1.75	1.23
1998–2002	1.32	1.33	1.08	1.03	0.86	0.70	0.76	0.78	0.84	0.95	1.14	1.32	1.47	1.61	1.59	1.61	1.64	1.67	1.31
2003–2007	1.18	1.28	1.17	1.06	0.88	0.78	0.78	0.86	0.90	0.97	1.14	1.32	1.49	1.64	1.76	1.73	1.63	1.53	1.35
2008–2012	1.12	1.23	1.20	1.18	0.92	0.68	0.76	0.84	0.89	0.92	1.10	1.27	1.46	1.58	1.75	1.84	1.68	1.54	1.32
2013–2016	1.08	1.22	1.05	1.03	0.84	0.69	0.65	0.71	0.80	0.86	1.07	1.22	1.37	1.56	1.68	1.72	1.73	1.55	1.25

**Table 2:** Top 10 cancers in Taiwan by sex between 1988 and 2016.

Sex	Rank	1988–1992				1993–1997				1998–2002			
		Cancer (ICD-O)	ASRw^*∗*^	%^*∗*^	Years^*∗*^	Cancer (ICD-O)	ASRw^*∗*^	%^*∗*^	Years^*∗*^	Cancer (ICD-O)	ASRw^*∗*^	%^*∗*^	Years^*∗*^
Male	1	Liver (155)	30.52	10.11	56.83	Liver (155)	38.73	17.56	58.48	Liver (155)	55.16	18.98	59.70
	2	Lung (162)	26.30	8.55	64.51	Lung (162)	32.10	14.68	66.40	Lung (162)	39.97	14.06	68.13
	3	Colorectum (153–154)	19.83	6.44	61.35	Colorectum (153–154)	26.92	12.30	63.28	Colorectum (153–154)	36.66	12.76	64.95
	4	Stomach (151)	19.50	6.22	63.91	Stomach (151)	18.95	8.59	65.75	Oral cavity and pharynx^*∗*^	26.44	9.26	52.43
	5	Oral cavity and pharynx^*∗*^	10.86	3.56	53.83	Oral cavity and pharynx^*∗*^	17.42	7.92	52.81	Stomach (151)	19.71	6.91	67.37
	6	Bladder (188)	7.04	2.22	64.48	Bladder (188)	10.64	4.75	72.14	Bladder (188)	17.46	6.23	73.23
	7	Esophagus (150)	5.90	1.90	62.82	Esophagus (150)	8.69	3.91	65.63	Esophagus (150)	10.79	3.76	67.32
	8	Prostate (185)	6.33	1.85	70.97	Prostate (185)	7.00	3.12	62.91	Prostate (185)	8.97	3.08	60.93
	9	Skin (173)	4.11	1.34	60.24	Skin (173)	5.53	2.51	62.61	Skin (173)	8.14	2.82	64.49
	10	Leukemia	3.48	1.24	38.21	Leukemia	4.45	2.09	41.16	Leukemia	5.57	1.88	46.14

Female	1	Cervix uteri (180)	26.27	7.95	53.18	Breast (174)	28.99	17.03	49.53	Breast (174)	39.32	18.62	50.57
	2	Breast (174)	20.95	6.54	49.05	Cervix uteri (180)	26.82	15.37	53.78	Colorectum (153–154)	28.99	13.00	63.96
	3	Colorectum (153–154)	16.76	4.81	60.20	Colorectum (153–154)	22.54	12.24	62.30	Cervix uteri (180)	23.69	11.01	55.22
	4	Lung (162)	11.91	3.37	62.14	Lung (162)	14.85	7.99	63.96	Liver (155)	21.81	9.61	64.62
	5	Stomach (151)	9.31	2.71	59.22	Stomach (151)	13.76	7.44	62.36	Lung (162)	19.55	8.73	65.57
	6	Liver (155)	8.72	2.52	59.61	Liver (155)	10.01	5.51	61.58	Stomach (151)	10.19	4.60	63.74
	7	Thyroid gland (193)	4.39	1.50	42.08	Thyroid gland (193)	6.43	3.95	43.08	Thyroid gland (193)	8.51	4.04	44.00
	8	Ovary (183)	4.11	1.31	47.23	Ovary (183)	5.28	3.09	18.16	Skin (173)	7.24	3.25	64.87
	9	Skin (173)	3.60	1.04	59.97	Skin (173)	5.20	2.83	62.41	Ovary (183)	6.63	3.08	49.83
	10	Leukemia	2.67	0.89	34.80	Leukemia	3.94	2.22	52.26	Corpus uteri (182)	5.73	2.61	52.86

Sex	Rank	2003–2007				2008–2012				2013–2016			
		Cancer (ICD-O)	ASRw^*∗*^	%^*∗*^	Years^*∗*^	Cancer (ICD-O)	ASRw^*∗*^	%^*∗*^	Years^*∗*^	Cancer (ICD-O)	ASRw^*∗*^	%^*∗*^	Years^*∗*^

Male	1	Liver (155)	56.99	17.82	61.14	Liver (155)	54.03	15.59	62.46	Colorectum (153-154)	52.63	15.71	65.92
	2	Lung (162)	42.65	13.82	69.16	Colorectum (153-154)	52.02	15.23	65.88	Liver (155)	47.56	14.01	63.46
	3	Colorectum (153-154)	43.02	13.67	65.82	Lung (162)	45.14	13.44	69.28	Lung (162)	44.23	13.33	68.34
	4	Oral cavity and pharynx^*∗*^	34.57	11.06	52.71	Oral cavity and pharynx^*∗*^	41.40	11.95	54.08	Oral cavity and pharynx^*∗*^	42.72	12.27	55.79
	5	Prostate	21.29	7.03	73.61	Prostate (185)	28.49	8.46	73.47	Prostate (185)	29.72	8.96	72.81
	6	Stomach	17.16	5.60	68.36	Stomach (151)	15.55	4.71	68.93	Esophagus (150)	14.44	4.26	58.67
	7	Esophagus (150)	11.34	3.60	59.13	Esophagus (150)	13.58	3.98	58.19	Stomach (151)	13.48	4.17	69.35
	8	Bladder (188)	10.60	3.43	68.28	Skin (173)	10.66	3.20	68.43	Skin (173)	11.62	3.57	70.34
	9	Skin (173)	9.00	2.89	66.16	Bladder (188)	9.51	2.85	69.53	Bladder (188)	9.03	2.78	69.91
	10	Leukemia	7.52	2.22	53.02	Non-hodgkin lymphoma	8.01	2.27	60.87	Non-hodgkin lymphoma	8.47	2.42	62.18

Female	1	Breast (174)	49.70	21.61	51.89	Breast (174)	63.14	23.89	53.65	Breast (174)	71.91	25.26	54.99
	2	Colorectum (153-154)	32.14	13.89	64.84	Colorectum (153-154)	36.13	14.40	65.68	Colorectum (153-154)	35.74	13.98	66.12
	3	Liver (155)	22.17	9.66	66.72	Lung (162)	25.48	10.16	66.22	Lung (162)	28.62	11.18	65.86
	4	Lung (162)	21.70	9.40	66.17	Liver (155)	21.85	8.64	68.78	Liver (155)	18.80	7.48	69.77
	5	Cervix uteri (180)	15.20	6.61	56.36	Thyroid gland (193)	13.45	4.75	47.37	Thyroid gland (193)	17.29	5.49	48.97
	6	Stomach (151)	9.34	4.08	65.20	Cervix uteri (180)	11.07	4.26	57.47	Corpus uteri (182)	13.48	4.81	55.18
	7	Thyroid gland (193)	9.46	3.98	46.35	Corpus uteri (182)	10.94	4.18	54.05	Skin (173)	8.59	3.57	71.68
	8	Skin (173)	7.72	3.37	67.29	Stomach (151)	8.69	3.51	66.67	Cervix uteri (180)	8.72	3.12	57.83
	9	Corpus uteri (182)	7.72	3.34	52.99	Skin (173)	8.43	3.47	70.14	Ovary (183)	9.09	3.03	52.75
	10	Ovary (183)	7.36	3.12	51.17	Ovary (183)	8.31	3.04	51.77	Stomach (151)	7.50	2.99	67.78

ASRw: age-standardized rate for the world population expressed as the number of incident cases per 100,000 individuals. %: the percentage of specific cancer incident cases in all incident cases. Years: the mean age of specific cancer incident cases. Oral caves and pharynx: the cancers of oral cavity and pharynx are classified to 140–146, 148–149.

## Data Availability

The data in this study were obtained from Taiwan Health Promotion Administration's (Ministry of Health and Welfare, MOHW) Interactive Online Cancer Registry Inquiry System, which is a public, free, and national database, and we performed in accordance with relevant national and international guidelines.
